# Association of single nucleotide polymorphisms in the *RAB5B* gene 3′UTR region with polycystic ovary syndrome in Chinese Han women

**DOI:** 10.1042/BSR20190292

**Published:** 2019-05-14

**Authors:** Jia Yu, Caifei Ding, Siqi Guan, Chenye Wang

**Affiliations:** 1 Reproductive Department, ZheJiang Chinese Medicine and Western Medicine Integrated Hospital/Hangzhou Red Cross Hospital, Hangzhou, P.R. China; 2 First Clinical Medical College, Beijing University of Chinese Medicine, No. 11 North Third Ring East Road, Beijing, P.R. China

**Keywords:** miR-24, miR-320, polycystic ovary syndrome, Ras-related protein Rab-5B, single nucleotide polymorphism

## Abstract

**Objective:** Previous genome-wide sequencing revealed that Ras-related protein Rab-5B (RAB5B) is a susceptible target in patients with polycystic ovary syndrome (PCOS).

**Methods:** Direct sequencing was performed to analyze the *RAB5B* gene rs1045435, rs11550558, rs34962186, rs705700, rs58717357, rs11171718, rs60028217, rs772920 loci genotypes in 300 PCOS patients and 300 healthy controls. The plasma microRNA (miRNA)-24, miR-320 levels were measured by reverse transcription fluorescent quantitative PCR (RT-qPCR).

**Results:** The risk of PCOS in C allele carriers of *RAB5B* gene rs1045435 locus was 3.91 times higher than that of G allele. The risk of PCOS in rs11550558 locus G allele was 4.09 times higher than A allele. The risk of PCOS in rs705700 locus C allele was 1.66 times greater than T allele. The risk of PCOS in rs11171718 locus A allele carrier was 3.84 times higher than G allele. The rs11550558 SNP was associated with PCOS risk only in those with age ≥ 31.1 years. And *RAB5B* gene rs11550558, rs1045435, and rs11171718 SNPs were significantly associated with PCOS risk only in subjects with BMI ≥ 23.8 kg/m^2^. We also found that the *RAB5B* gene rs1045435 SNP was associated with plasma miR-24 levels. The *RAB5B* gene rs11550558, rs705700, rs11171718 SNPs were correlated with plasma miR-230 levels.

**Conclusion:** The single nucleotide polymorphisms of the rs1045435, rs11550558, rs705700, and rs11171718 loci of the *RAB5B* gene are associated with PCOS risk. The rs1045435 locus is likely an miR-24 binding site, while rs11550558, rs705700, and rs11171718 loci may be miR-320 binding sites.

## Introduction

Polycystic ovary syndrome (PCOS) is an endocrine disorder with a complex pathogenesis and a high degree of heterogeneity in clinical manifestations. Signs and symptoms of PCOS include irregular or no menstrual periods, high androgens induced symptoms such as excess hairy and acne, bilateral or unilateral ovarian polycystic changes, difficulty getting pregnant, obesity, insulin resistance and type 2 diabetes [1,2].

PCOS is a common endocrine and metabolic disorder that affects women of childbearing age. Its etiology and pathogenesis are related not only to genetic factors, but also to environmental factors [3–6]. Recent studies have shown that the etiology of PCOS is likely correlated with genes involved in androgen elevated, insulin resistance and insulin-regulating genes, as well as those involved in metabolism, steroid hormones and glandular hormone biosynthesis [7–10].

The *Ras-related protein Rab-5B* (*RAB5B*) gene is located on the 12q13.2 chromosome, which encodes a small GTPase involved in cellular phagocytosis, mucosal transport and its receptor expression [11]. These small GTPases mainly affect multiple cellular pathways in the ovarian follicular cells and amplify these signals, which result in corresponding clinical manifestations and endocrine changes in PCOS patients [12,13]. A previous study implicated that the expression of *RAB5B* gene in skeletal muscle is increased 2–3 times in subjects with insulin resistance [14]. Consistently, *RAB5B* gene could increase insulin resistance via reducing the function of glucose transporter-4 (GLUT4) on the plasma mucosal surface [15]. Thus, the *RAB5B* gene might result in the onset of PCOS by affecting insulin function and androgen levels.

In the present study, we aimed to analyze the correlates of the single nucleotide polymorphism of the *RAB5B* gene on the risk of PCOS. We screened for possible sensitive SNPs from the 3′ URT region of the *RAB5B* gene. Firstly, eight SNPs were selected by the variation Viewer function in NCBI database with 1000 Genomes MAF above 0.05, which are rs1045435, rs11550558, rs34962186, rs705700, rs58717357, rs11171718, rs60028217, rs772920. We systematically analyzed the correlation between these SNPs and the risk of PCOS in Chinese Han population by a case–control study.

## Materials and methods

### Ethical statement

The present study was approved by the Institutional Ethics Committees of the following participating institutes: ZheJiang Chinese Medicine and Western Medicine Integrated Hospital/Hangzhou Red Cross Hospital, and First Clinical Medical College at Beijing University of Chinese Medicine. All the participants signed their informed written consent. The present study conforms to the principles outlined in the World Medical Association Declaration of Helsinki.

### Study participants

The main study participants comprised 300 cases with PCOS, who were recruited from ZheJiang Chinese Medicine and Western Medicine Integrated Hospital/Hangzhou Red Cross Hospital, and First Clinical Medical College at Beijing University of Chinese Medicine during February 2016 and October 2018. The diagnosis of PCOS was based on two of three criteria described in Rotterdam’s 2003: irregular and/or no ovulation, elevated androgen levels, and ovarian cysts. And subjects with other etiology were excluded from the present study, including congenital adrenal hyperplasia, androgen-secreting tumors, and Cushing’s syndrome related clinical and/or biochemical signs [16]. The irregular or no ovulation usually characterized by oligomenorrhea (<9 cycles per year) or amenorrhea, thus those individuals are under higher risk of infertility. Clinically, hyperandrogenemia is manifested as hirsutism, acne and androgenetic alopecia, and/or total testosterone levels above 55.07 ng/dl (1.91 nmol/l). In the present study, the diagnosis criteria of PCOS is the presence of 12 or more follicles in each ovary, 2 ± 9 mm in diameter, and/or an increase in ovarian volume (>10 ml). PCOS patients with congenital adrenal hyperplasia, Cushing’s syndrome, and androgen-secreting tumors were not included in the study. In addition, patients with diabetes and glucose intolerance as well as PCOS caused by metabolic disorders were also excluded from the present study. Healthy women without any endocrine dysfunction and any other diseases were recruited as controls. All participants in the control group were normal women with normal menstrual cycles, normal ovulation, and no endocrine dysfunction associated with PCOS. The follicular growth of both cases and controls were monitored for 3 months before transvaginal ultrasound detection. All subjects have not received hormonal therapy within 3 months before the present study. We recorded the subject’s age of menarche (AAM) by face-to-face interview and calculated the body mass index (BMI). Peripheral blood was collected from day 3 to day 5 of the menstrual cycle, from all subjects with limosis in the morning. EDTA anticoagulated peripheral blood sample was immediately centrifuged, and then the serum was isolated and stored at −80°C until use. The level of follicle stimulating hormone (FSH), luteinizing hormone (LH), total testosterone, prolactin, thyroid stimulating hormone (TSH), and estradiol (E2) was determined by radioimmunoassay (RIA; Beijing North Institute of Biological Technology of China and the CIS Company of France), the intra- and inter-assay coefficient of variation was controlled within 10%.

### Genotyping

We collected leukocytes from serum samples of all participants with limosis and extracted genomic DNA using QIAamp DNA Blood Mini Kit 250 (QIAGEN, Germany, Hilden). The experimental procedures were strictly following the standard operating procedures according to the manufacturer, and the extracted genomic DNA was preserved at −80°C until use. Polymerase Chain Reaction (PCR) was performed according to the PCR primer information of SNPs (Table 1). The total volume of the PCR was 50 μl, including 100 ng of extracted genomic DNA, 10 pmol/μl of Forward and Reverse primers, 100 μM of dNTP, and 1 U/μl of Taq DNA polymerase, 2 mM of Mg^2+^, and add sterile water to the final volume. The PCR conditions were: 94°C, 5 min; 98°C, 10 s; 58°C, 15 s; 72°C, 2 min, a total of 30 cycles followed by a final extension at 72°C for 5 min. All PCR products were sequenced by BioSune Biotechnology Co., Ltd. and sequence data was analyzed using ChromasPro (version 1.42; Technelysium Pty Ltd) software.

**Table 1:** PCR primer sequences for *RAB5B* gene SNPs

SNPs	PCR sequencing primers (5′–3′)	Product length
rs1045435	Forward primer: GGCCTTATGAGGCAGGTGAG;	112 bp
	Reverse primer: TAGGAGGTGGACGTCAGAGG	
rs11550558	Forward primer: AACCTCCATCCCTACCCCTC;	124 bp
	Reverse primer: TGGTGTTTGTTGCTGAAGCG	
rs34962186	Forward primer: GTGTAGGGTATGGTCTGTGGA;	113 bp
	Reverse primer: GCCCAAAGATCCACAGACCA	
rs705700	Forward primer: TGTAGCTTCCACCTCAACGG;	85 bp
	Reverse primer: GACTGTGGTGGACTGAAGGG	
rs58717357	Forward primer: AGGCCTGTCTCTGTCAACTT;	73 bp
	Reverse primer: TGAAGAAACGAGGCGGAAGG	
rs11171718	Forward primer: CCTTCCGCCTCGTTTCTTCA;	107 bp
	Reverse primer: AGCCTCCTTCCTAATAGTGTCA	
rs60028217	Forward primer: AGGTTGGGCATTAGCCTTCT;	78 bp
	Reverse primer: GGCTCTTAAGTGTTTGGAAATGGA	
rs772920	Forward primer: GGGGTGTTAAGCTGCCATTG;	281 bp
	Reverse primer: GGGCTAGTCGAATTTCTCCCC	

Abbreviation: SNPs, single nucleotide polymorphisms.

### Reverse transcription-polymerase chain reaction for detecting plasma miRNAs levels

Total RNA was extracted using TRIzol reagent (GIBCO BRL, Rockville, MD) according to the manufacturer’s instruction, and finally added in 60 μl of pre-warmed (95°C) nuclease-free water. The cDNA was synthesized by reverse transcription reaction using Taqman MicroRNA Reverse Transcription Kit (Applied Biosystems). The plasma levels of miR-24, miR-320 were then determined by the miScript SYBR Green PCR Kit with U6 as a control. The miR-24 primer sequence was: Forward, 5′-GCG CCG GTG ACT CAG TAC AGC-3′; Reverse, 5′-GCG CCG GTG ACT CAG TAC AGC-3′. The miR-320 primer sequence was: Forward, 5′-ACT ATG GAA AAG CTG GGT TGA GAG-3′; Reverse, 5′-ATT CGT TGA GAG ATC ACA AGC GT-3′. The U6 primer sequence was: Forward, 5′-GCC TGC TCC GGC AGC AC-3′; Reverse, 5′-GAG GTA TTC GCA CCA GAG GA-3′. The PCR conditions were: pre-denaturation at 95°C for 60 s, followed by 95°C, 15 s; 60°C, 30 s; 72°C, 30 s; and 40 cycles followed by 95°C, 15 s; 60°C, 30 s; 95°C, 15 s for analyzing the melting curve. Three replicates were prepared for each sample, and the levels of miR-24 and miR-320 relative to the reference U6 were calculated by 2^−ΔΔCT^.

### Statistical analysis

Statistical analyses were performed using SPSS 21.0 (SPSS Inc, Chicago, IL). Continuous variable data were expressed as mean ± SD, categorical variables were expressed as [*n* (%)]. Fisher’s Exact Test was applied to compare the genotype distribution of *RAB5B* gene SNPs between PCOS and control groups. To determine the association between RAB5B gene SNPs and PCOS risk, χ^2^ test was performed to test whether the genotype distribution is consistent with Hardy–Weinberg equilibrium (HWE), based on the distribution of allele frequencies and genetic models (additive, dominant and recessive models), and adjusted for age, BMI. Odds ratio (OR) and 95% confidence interval (CI) were calculated using unconditional logistic regression analysis. The interaction of RAB5B gene SNPs loci was analyzed by a multi-factor dimensionality reduction (MDR) method. All tests were two-tailed, with *P* < 0.05 was considered as significant differences.

## Results

### General characteristics of study subjects

The general clinical data of 300 PCOS patients and 300 control subjects are shown in Table 2. There was no significant difference in age, FSH level and AAM between PCOS patients and healthy controls (*P*>0.05). However, the BMI, LH, total testosterone, Prolactin, TSH and E2 were significantly increased in PCOS patients compared with those in the control group (*P* < 0.001).

**Table 2:** General characteristics of PCOS patients and healthy controls

Parameters	PCOS (*n* = 300)	Control (*n* = 300)	*P*
Age (years, mean ± SD)	30.72 ± 6.81	31.56 ± 7.04	0.11
BMI (kg/m^2^, mean ± SD)	25.01 ± 3.70	22.64 ± 3.60	**<0.001**
FSH (IU/l, mean ± SD)	7.31 ± 3.19	7.35 ± 2.36	0.67
LH (IU/l, mean ± SD)	15.47 ± 5.858	4.9 ± 2.15	**<0.001**
Total testosterone (ng/ml, mean ± SD)	0.94 ± 0.42	0.52 ± 0.19	**<0.001**
Prolactin (ng/ml, mean ± SD)	17.18 ± 8.87	14.65 ± 3.13	**<0.001**
TSH (μIU/ml, mean ± SD)	2.64 ± 0.83	1.52 ± 0.42	**<0.001**
E_2_ (pg/ml, mean ± SD)	222.30 ± 77.41	166.56 ± 73.49	**<0.001**
AAM (years, mean ± SD)	14.27 ± 1.52	14.21 ± 2.29	0.53

Abbreviations: AAM, age at menarche; BMI, body mass index; E_2_, estradiol; FSH, follicle stimulating hormone; LH, luteinizing hormone; PCOS, polycystic ovary syndrome; TSH, thyroid stimulating hormone.

### Association of *RAB5B* gene SNPs with PCOS risk

The distribution of *RAB5B* gene SNPs loci genotypes and allelic frequency in the PCOS and control groups is shown in Table 3. The genotype frequencies of the SNPs of the *RAB5B* gene were consistent with the Hardy–Weinberg equilibrium (*P*>0.05). The risk of PCOS was significantly increased in heterozygous, homozygous, dominant and recessive models of rs1045435 locus (*P* < 0.05). And the risk of PCOS in C allele carriers was 3.91 times higher than G allele carriers (95% CI: 2.16–7.17, *P*<0.001). The risk of PCOS was significantly increased in heterozygous, homozygous, dominant and recessive models of rs11550558 locus (*P*<0.05), and the risk of PCOS in G allele carriers was 4.09 times higher than A allele carriers (95% CI: 2.22–7.64, *P*<0.001). While, the genotypes and allele frequencies of rs34962186, rs58717357, rs60028217, and rs772920 were not significantly different between the PCOS and the control groups (*P*>0.05). The risk of PCOS was higher in the rs705700 locus homozygous, dominant model, and recessive model (*P*<0.05), and the risk in C allele carrier was 1.66 times higher than those of the T allele (95% CI: 1.28–2.14, *P* < 0.001). Similarly, the risk of PCOS was significantly increased in heterozygous, homozygous, dominant and recessive models of rs11171718 locus (*P*<0.05), and the risk in A allele carrier was 3.84 times higher than those of G allele (95% CI: 2.43–6.11, *P*<0.001).

**Table 3:** Genotype frequencies of *RAB5B* gene SNPs loci in PCOS and controls

	PCOS (*n* = 300)	Control (*n* = 300)	HWE *P*	OR (95%CI)*	P*
**rs1045435**					
GG	255 (85.00%)	285 (95.00%)	0.08	Reference	
GC	32 (10.67%)	14 (4.67%)		2.56 (1.28–5.16)	**0.006**
CC	13 (4.33%)	1 (0.33%)		14.53 (1.97–299.74)	**0.001**
Additive model				1.12 (0.88–1.42)	0.38
Dominant model				3.35 (1.76–6.46)	**<0.001**
Recessive model				13.54 (1.84–279.24)	**0.003**
G	542 (90.33%)	584 (97.33%)		Reference	
C	58 (9.67%)	16 (2.67%)		3.91 (2.16–7.17)	**<0.001**
**rs11550558**					
AA	251 (83.67%)	286 (95.33%)	0.06	Reference	
AG	41 (13.67%)	13 (4.33%)		3.59 (1.81–7.24)	**<0.001**
GG	8 (2.67%)	1 (0.33%)		9.12 (1.15–195.71)	**0.03**
Additive model				1.14 (0.90–1.45)	0.30
Dominant model				3.99 (2.08–7.77)	**<0.001**
Recessive model				8.19 (1.04–175.76)	**0.04**
A	543 (90.50%)	585 (97.50%)		Reference	
G	57 (9.50%)	15 (2.50%)		4.09 (2.22–7.64)	**<0.001**
**rs34962186**					
TT	261 (87.00%)	266 (88.67%)	0.06	Reference	
TC	34 (11.33%)	31 (10.33%)		1.12 (0.65–1.93)	0.77
CC	5 (1.67%)	3 (1.00%)		1.70 (0.35–9.05)	0.71
Additive model				1.02 (0.80–1.30)	0.92
Dominant model				1.17 (0.70–1.96)	0.62
Recessive model				1.68 (0.35–8.93)	0.72
T	556 (92.67%)	563 (93.83%)		Reference	
C	44 (7.33%)	37 (6.17%)		1.20 (0.75–1.94)	0.49
**rs705700**					
TT	145 (48.33%)	173 (57.67%)	0.08	Reference	
TC	94 (31.33%)	102 (34.00%)		1.10 (0.76–1.60)	0.67
CC	61 (20.33%)	25 (8.33%)		2.91 (1.69–5.04)	**<0.001**
Additive model				1.19 (0.90–1.58)	0.23
Dominant model				1.46 (1.04–2.04)	**0.03**
Recessive model				2.80 (1.67–4.76)	**<0.001**
T	384 (64.00%)	448 (74.67%)		Reference	
C	216 (36.00%)	152 (25.33%)		1.66 (1.28–2.14)	**<0.001**
**rs58717357**					
GG	234 (78.00%)	241 (80.33%)	0.14	Reference	
GC	61 (20.33%)	53 (17.67%)		1.19 (0.77–1.82)	0.48
CC	5 (1.67%)	6 (2.00%)		0.86 (0.22–3.22)	0.80
Additive model				1.03 (0.80–1.32)	0.86
Dominant model				1.15 (0.76–1.74)	0.55
Recessive model				0.83 (0.22–3.11)	0.76
G	529 (88.17%)	535 (89.17%)		Reference	
C	71 (11.83%)	65 (10.83%)		1.11 (0.76–1.60)	0.65
**rs11171718**					
GG	231 (77.00%)	274 (91.33%)	0.08	Reference	
GA	43 (14.33%)	24 (8.00%)		2.13 (1.22–3.73)	**0.007**
AA	26 (8.67%)	2 (0.67%)		15.42 (3.51–95.02)	**<0.001**
Additive model				1.19 (0.93–1.52)	0.18
Dominant model				3.15 (1.89–5.26)	**<0.001**
Recessive model				14.14 (3.22–87.00)	**<0.001**
G	505 (84.17%)	572 (95.33%)		Reference	
A	95 (15.83%)	28 (4.67%)		3.84 (2.43–6.11)	**<0.001**
**rs60028217**					
TT	244 (81.33%)	251 (83.67%)	0.07	Reference	
TA	51 (17.00%)	44 (14.67%)		1.19 (0.75–1.90)	0.50
AA	5 (1.67%)	5 (1.67%)		1.03 (0.26–4.15)	0.97
Additive model				1.03 (0.81–1.32)	0.86
Dominant model				1.18 (0.76–1.83)	0.52
Recessive model				\	\
T	539 (89.83%)	546 (91.00%)		Reference	
A	61 (10.17%)	54 (9.00%)		1.14 (0.77–1.71)	0.56
**rs772920**					
CC	185 (61.67%)	177 (59.00%)	0.06	Reference	
CG	91 (30.33%)	99 (33.00%)		0.88 (0.61–1.27)	0.53
GG	24 (8.00%)	24 (8.00%)		0.96 (0.50–1.82)	0.89
Additive model				0.96 (0.73–1.25)	0.79
Dominant model				0.90 (0.64–1.26)	0.56
Recessive model				\	\
C	461 (76.83%)	453 (75.50%)		Reference	
G	139 (23.17%)	147 (24.50%)		0.93 (0.71–1.22)	0.64

* Adjusted for age and BMI. Abbreviations: CI, confidence interval; HWE, Hardy–Weinberg equilibrium; OR, odds ratio; PCOS, Polycystic ovary syndrome.

### Haplotype analysis for *RAB5B* gene SNPs

**Figure 1:**
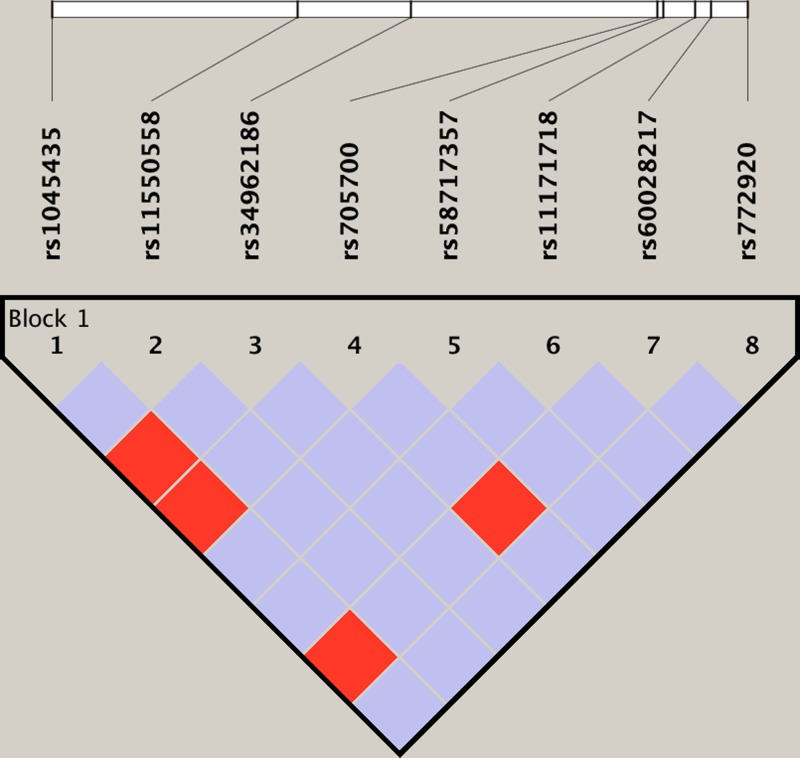
Linkage disequilibrium map of the *RAB5B* gene SNPs

Two haploids were formed in the *RAB5B* gene rs1045435, rs11550558, rs34962186, rs705700, rs58717357, rs11171718, rs60028217, and rs772920 loci, they are CGCCCAAG and GACCGGTA, respectively. The analysis revealed that CGCCCAAG haplotype is a high risk of PCOS (OR = 3.23, 95% CI: 1.76–5.67, *P* = 0.005), as shown in Figure 1.

### Effects of age factor on the relationship between *RAB5B* gene SNPs and PCOS risk

The mean age of all subjects enrolled in the current study was 31.1 years. We found that the risk of PCOS in subjects with rs1045435 mutation (GC/CC) was significantly higher than wild-type subjects (GG) at ages both <31.1 years and ≥31.1 years (*P*<0.05) (Table 4). In subjects with age < 31.1 years, there was no statistically significant difference in the risk of PCOS between subjects with rs11550558 mutation (GC/CC) and those with wild-type (GG) (*P*>0.05). However, in subjects with age > 31.1 years, the risk of PCOS in subjects with rs11550558 mutation (GC/CC) was 9.25 times higher than those of wild-type (GG) (95% CI: 2.97–32.10, *P*<0.001) (Table 4). Moreover, age factor has no impact in the correlation between rs705700 locus SNPs and PCOS risk (*P*>0.05) (Table 4). However, in subjects with rs11171718 locus mutation (GA/AA), the risk was significantly higher than those of wild type (GG) at ages both <31.1 and ≥31.1 years (*P*<0.05) (Table 4).

**Table 4:** Distribution of genotype frequencies of *RAB5B* gene SNPs in PCOS and control subjects with different ages

	PCOS (*n* = 300)	Control (*n* = 300)	OR (95% CI)*	P*
**<31.1 years**				
rs1045435				
GG	136 (83.44%)	142 (93.42%)	Reference	
GC/CC	27 (15.56%)	10 (6.58%)	2.82 (1.25–6.50)	**0.01**
rs11550558				
AA	142 (87.12%)	142 (93.42%)	Reference	
AG/GG	21 (12.88%)	10 (6.58%)	2.10 (0.90–4.98)	0.09
rs705700				
TT	82 (50.61%)	92 (60.53%)	Reference	
TC/CC	81 (49.69%)	60 (39.47%)	1.52 (0.95–2.43)	0.09
rs11171718				
GG	120 (73.62%)	135 (89.47%)	Reference	
GA/AA	43 (26.38%)	16 (10.53%)	3.05 (1.57–5.97)	**0.001**
**≥31.1 years**				
rs1045435				
GG	119 (86.86%)	143 (96.62%)	Reference	
GC/CC	18 (13.14%)	5 (3.38%)	4.33 (1.46–13.76)	**0.005**
rs11550558				
AA	109 (79.56%)	144 (97.30%)	Reference	
AG/GG	28 (20.44%)	4 (2.70%)	9.25 (2.97–32.10)	**<0.001**
rs705700				
TT	63 (45.99%)	81 (54.73%)	Reference	
TC/CC	74 (54.01%)	67 (45.27%)	1.42 (0.87–2.33)	0.18
rs11171718				
GG	111 (81.02%)	138 (93.24%)	Reference	
GA/AA	26 (18.98%)	10 (6.76%)	3.23 (1.42–7.53)	**0.003**

* Adjusted for BMI. Abbreviations: CI, confidence interval; OR, odds ratio; PCOS, polycystic ovary syndrome.

### Effects of BMI on the relationship between *RAB5B* gene SNPs and PCOS risk

The mean BMI for all subjects enrolled in the current study was 23.8 kg/m^2^. We observed that in subjects with BMI <23.8 kg/m^2^, the risk of PCOS in subjects with rs1045435, rs11550558, rs705700, and rs11171718 mutations was not significant difference from those of wild-type (*P*>0.05) (Table 5). Among subjects with BMI≥23.8 kg/m^2^, the risk of PCOS in those with rs11550558, rs1045435 and rs11171718 mutations was significantly higher than that in wild type (*P*<0.05), however, there was no significant difference in PCOS risk between rs705700 locus mutation and wild-type subjects (*P*>0.05) (Table 5).

**Table 5:** Distribution of genotype frequencies of *RAB5B* gene SNPs in PCOS and control subjects with different BMI

	PCOS (*n* = 300)	Control (*n* = 300)	OR (95% CI)*	P*
**<23.8 kg/m^2^**				
rs1045435				
GG	68 (98.55%)	136 (96.45%)	Reference	
GC/CC	1 (1.45%)	5 (3.55%)	0.40 (0.02–3.63)	0.68
rs11550558				
AA	66 (95.65%)	134 (95.04%)	Reference	
AG/GG	3 (4.35%)	7 (4.96%)	0.87 (0.17–3.90)	0.84
rs705700				
TT	32 (46.38%)	82 (58.16%)	Reference	
TC/CC	37 (53.62%)	59 (41.84%)	1.61 (0.87–2.99)	0.14
rs11171718				
GG	64 (92.75%)	127 (90.07%)	Reference	
GA/AA	5 (7.25%)	14 (9.93%)	0.71 (0.21–2.23)	0.70
**≥23.8 kg/m^2^**				
rs1045435				
GG	187 (80.95%)	149 (93.71%)	Reference	
GC/CC	44 (19.05%)	10 (6.29%)	3.51 (1.64–7.71)	**0.001**
rs11550558				
AA	185 (80.09%)	152 (95.60%)	Reference	
AG/GG	46 (19.91%)	7 (4.40%)	5.40 (2.26–13.51)	**<0.001**
rs705700				
TT	113 (48.92%)	91 (57.23%)	Reference	
TC/CC	118 (51.08%)	68 (42.77%)	1.40 (0.91–2.14)	0.13
rs11171718				
GG	167 (72.29%)	147 (92.45%)	Reference	
GA/AA	64 (27.71%)	12 (7.55%)	4.70 (2.35–9.57)	**<0.001**

* Adjusted for age. Abbreviations: CI, confidence interval; OR, odds ratio; PCOS, polycystic ovary syndrome.

### MDR analysis of RAB5B gene SNPs loci gene–gene interaction

**Figure 2:**
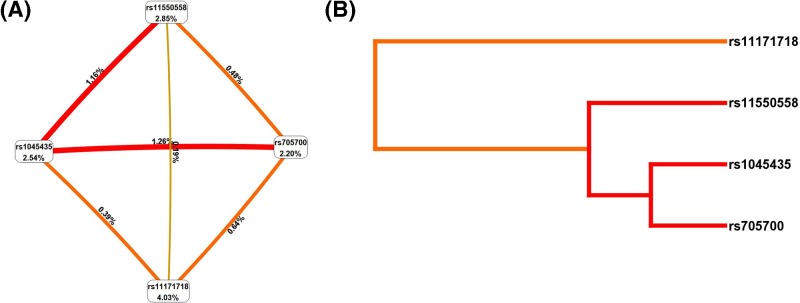
MDR analysis of *RAB5B* gene rs1045435, rs11550558, rs705700, rs11171718 loci gene–gene interaction (**A**) Circle graph, the percentage at the bottom of each polymorphism represented entropy of it, and the percentage on each line represented in interaction percentage of entropy between two polymorphisms. (**B**) Dendogram, red lines represent synergistic interactions, with darker colors representing stronger interactions and orange colors representing weaker interactions.

We performed multi-factor dimensionality reduction (MDR) to analyze the interaction of *RAB5B* gene SNPS rs1045435, rs11550558, rs705700, rs11171718 with the risk of PCOS, and adjusted for age and BMI factors by logistic regression analysis. We found that the interaction between rs1045435 and rs705700 was the best model with consistency of 10/10 (Table 6), and the interaction between rs1045435 and rs705700 loci was the most robust (Figure 2). Subjects with both rs1045435 CC genotype and rs705700 CC genotype had a higher risk of PCOS (OR = 4.12, 95% CI: 2.23–7.31, *P*<0.001).

**Table 6:** *RAB5B* gene rs1045435, rs11550558, rs705700, rs11171718 loci gene–gene interaction

Model	Bal. Acc. CV testing	CV consistency	*P*
rs11171718	0.54	5/10	0.65
rs1045435, rs705700	0.65	10/10	**<0.001**
rs1045435, rs11550558, rs705700	0.58	9/10	0.06
rs1045435, rs11550558, rs705700, rs11171718	0.53	7/10	0.08

Abbreviations: Bal. Acc., balanced accuracy; CV, cross-validation.

### MDR analysis of the interaction between *RAB5B* gene SNP loci gene and environmental factors

**Figure 3:**
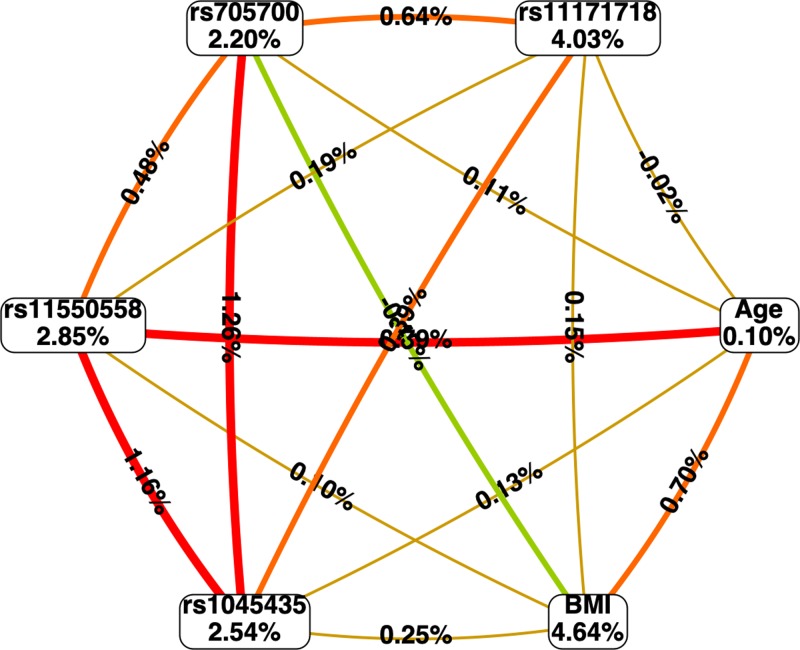
Circle graph showing the MDR analysis of interactions between RAB5B gene rs1045435, rs11550558, rs705700, rs11171718 loci gene–environmental interaction The percentage at the bottom of each polymorphism represented entropy of it, and the percentage on each line represented in interaction percentage of entropy between SNP and environment factors.

We used MDR to analyze the association between *RAB5B* gene rs1045435, rs11550558, rs705700, rs11171718 loci and age, BMI with PCOS. The results showed that there was a strong interaction between rs11550558 and BMI (Figure 3), while subjects carrying rs11550558 GG genotype and a BMI more than 23.8 kg/m^2^ had a higher risk of PCOS (OR = 2.24, 95% CI: 1.13–4.16, *P* = 0.005).

### The levels of plasma miR-24 and miR-320 in PCOS patients and controls

**Figure 4:**
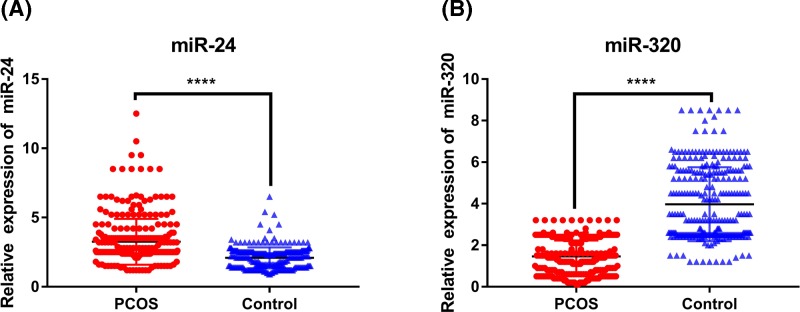
The plasma miR-24 and miR-320 levels in PCOS and controls ****P<0.001.

The levels of miR-24 and miR-320 in plasma of PCOS patients and control subjects were determined by RT-PCR. We found that the level of miR-24 in plasma of PCOS patients was significantly higher than that of controls, while the level of miR-320 was significantly lower than that of controls, with both differences were statistically significant (*P* < 0.001) (Figure 4).

### Correlations of *RAB5B* gene SNPs with levels of plasma miR-24, miR-230

**Figure 5:**
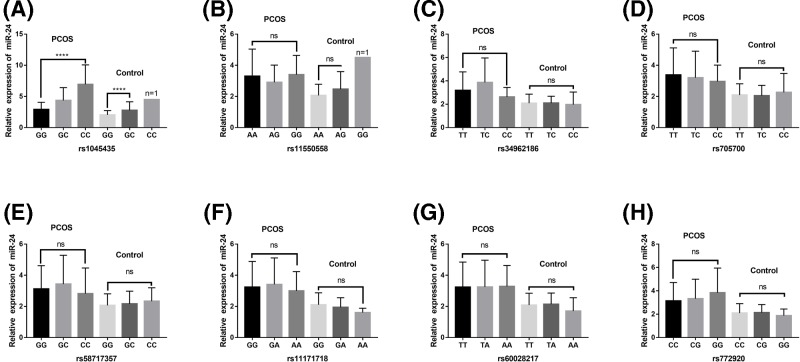
Associations of plasma miR-24 level with *RAB5B* gene 3′UTR region SNPs in both PCOS and controls Plasma miR-24 levels in subjects with different genotypes at (**A**) rs1045435; (**B**) rs11550558; (**C**) rs34962186; (**D**) rs705700; (**E**) rs58717357; (**F**) rs11171718; (**G**) rs60028217; (**H**) rs772920. ****P<0.001. Abbreviations: ns, no statistical difference; PCOS, polycystic ovary syndrome.

The plasma miR-24 level of *RAB5B* gene rs1045435 mutation in PCOS patients was significantly higher than that of wild type (*P*<0.001). Consistently, in controls there is only one case of homozygotes, and the plasma miR-24 levels were significantly higher in heterozygous than wild type (*P* < 0.001). In both PCOS and control groups, the plasma level of miR-24 was not significant difference in different genotypes of rs11550558, rs34962186, rs705700, rs58717357, rs11171718, rs60028217, rs772920 loci (*P*>0.05) (Figure 5).

**Figure 6:**
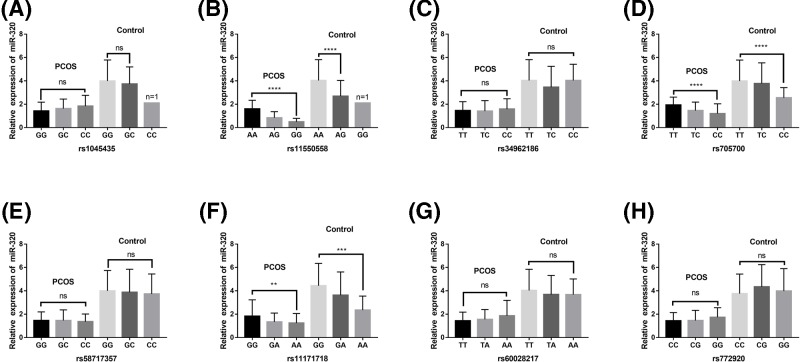
Associations of plasma miR-320 level with *RAB5B* gene 3′UTR region SNPs in both PCOS and controls Plasma miR-320 levels in subjects with different genotypes at (**A**) rs1045435; (**B**) rs11550558; (**C**) rs34962186; (**D**) rs705700; (**E**) rs58717357; (**F**) rs11171718; (**G**) rs60028217; (**H**) rs772920. ****P<0.0001. ***P<0.001. **P<0.01. Abbreviations: ns, no statistical difference; PCOS, polycystic ovary syndrome.

In contrast, in both PCOS and control groups, the plasma miR-230 levels of *RAB5B* gene rs11550558, rs705700, rs11171718 mutants were significantly lower than those in the wild type (*P*<0.05). However, there were no significant difference in plasma miR-230 levels between subjects with different genotypes of rs1045435, rs34962186, rs58717357, rs60028217, and rs772920 (*P*>0.05) (Figure 6).

## Discussion

PCOS is a highly heterogeneous and multi-gene mediated complex disease, it has been drawn a considerable attention by multiple studies, however, the precise underlying mechanisms remain elusive [1,17,18]. A growing body of evidence suggests that PCOS is affected by a multi-gene interactions together with environmental factors [19,20]. In the last decades, genome-wide association research (GWAS) has become a new approach to analyze the influence of genetic factors on the pathogenesis of PCOS. Using GWAS method for the first time, previous studies have reported three susceptibility genes in the case–control study of Chinese Han PCOS patients [21]. Further, also by GWAS, Shi et al. [22] revealed some new susceptibility genes after expanding the sample size. Although some genes were verified in different populations, the exact functions of these genes were still unclear and controversial. It is obviously necessary to investigate the function of a single gene. Therefore, in the present study, we selected one of the *RAB5B* genes as a target, aiming to explore its role in the pathogenesis of PCOS through a case–control study.

We studied eight SNPs in the 3′UTR region of *RAB5B* gene, which are rs1045435, rs11550558, rs34962186, rs705700, rs58717357, rs11171718, rs60028217, rs772920. We found that the risk of PCOS was increased significantly in subjects carrying the C allele at the rs1045435 locus of *RAB5B* gene, exclusion the interferences of age and BMI factors. In order to assess the effects of age and BMI, we further performed multiple analyses to age and BMI factors. We grouped all data by the median of age and the median of BMI, and found that only in subjects with age ≥31.1 years, the risk of PCOS was associated with rs11550558 SNP. And only in subjects with BMI≥23.8 kg/m^2^, the risk of PCOS was associated with rs11550558, rs1045435 and rs11171718 loci SNPs. These results indicate that for older female subjects, the risk of PCOS is higher in rs11550558 G allele carriers, and for obese (BMI ≥ 23.8 kg/m^2^) Chinese Han women, the risk of PCOS is higher in rs11550558 G allele carriers, the rs1045435 C allele carriers or the rs11171718 locus A allele carriers. Hence, it is necessary for these patients to take precautions in advance. Using MDR analysis, we revealed that rs1045435 had robust interaction with rs705700, suggesting that a higher risk of PCOS might we exist for those carrying both rs1045435 C allele and rs705700 C allele. These results also provided clinical significance for PCOS prevention.

**Figure 7:**
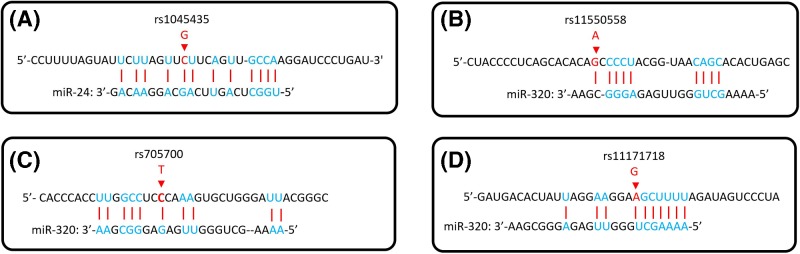
Analyses of miR-24, miR-230 binding sites in *RAB5B* gene 3′UTR region The red arrow indicates the base of the SNPs loci mutations in the 3′UTR region of *RAB5B* gene.

The 3′UTR region contains binding sites for microRNAs (miRNAs). To further investigate whether miRNAs play a major role in the correlation between SNPs in the 3′UTR region of *RAB5B* gene and PCOS risk, we conducted TargetScan 3.1 analysis (http://www.targetscan.org/mamm_31/), and found two miRNAs, miR-24 and miR-320. The predicted results showed that the binding site of miR-24 to the 3′UTR region of *RAB5B* gene was located at rs1045435 (Figure 7A), while miR-320 binding site to the 3′UTR region of the *RAB5B* gene was located at the rs11550558 (Figure 7B), rs705700 (Figure 7C), and rs11171718 loci (Figure 7D). The Smads family proteins play a key role in the transduction of transforming growth factor beta (TGF-β) signaling from cell surface to the nucleus, and different Smads proteins mediate signal transduction by different TGF-β family members [23,24]. Previous studies have shown that miR-24 might inhibit the production of estradiol by suppressing the expression of Smad protein, which causes inhibition of the TGF-β signaling pathway [25]. In the present study, we found that the level of miR-24 in plasma of PCOS patients was significantly higher than that of the control group. However, Sorensen et al. [26] reported that the level of miR-24-3p in follicular fluid of PCOS patients was lower than that of the control group, which is inconsistent with the results of the present study. We considered it may be related to ethnic differences. Further, the regulation of miRNAs expression *in vivo* is very complicated, and the levels of miRNA might be affected by multiple factors. Meanwhile, we found that the plasma miR-24 level was associated with rs1045435 SNP, and the C allele carrier had both higher risk for PCOS and plasma miR-24 levels. For the underlying mechanism of the relationship between plasma miR-24 levels and rs1045435 SNPs observed in the current study, we speculate that the rs1045435 SNP is associated with the risk of PCOS, while the plasma miR-24 levels are higher in patients with PCOS; however, the higher levels of plasma miR-24 in PCOS patients are not determined by the rs1045435 SNP.

Furthermore, in consistent with Sorensen et al. [27] and Sang et al. [28], we found that the level of miR-320 in patients with PCOS was significantly lower than that in the control group. Our further studies showed that the level of miR-320 in plasma was correlated with s11550558, rs705700, and rs11171718 SNPs. Therefore, we speculated that the diverse alleles of SNPs may have different binding efficiency to miR-320, which lead to different regulation efficiency of *RAB5B* gene expression miR-320, and finally cause the difference in the risk of POCS in participants. However, there is no direct evidence in the current study to support this speculation, and further research is needed.

There were several limitations in the present study. Firstly, it is difficult to recruit large samples of PCOS patients. Therefore, the sample size is fairly small for the rare mutant patients, which may affect the objectivity of our results. Secondly, we did not have *ex vivo* study to verify the precise mechanism underlying regulation of *RAB5B* gene expression by miR-24 and miR-320, which is one of the difficulties. In addition, we considered that other related genes may also have certain effects on the etiology of PCOS, such as genes involved in androgen elevation, insulin resistance and insulin-regulation, metabolism, steroid hormones, and genes that promote glandular hormone biosynthesis. It is valuable to analyze the interaction between multiple genes, which may represent one of the possible directions for future study. Another limitation of the current study is that we did not analyze the association between these SNPs and clinical symptoms.

## Conclusions

Here, we report that *RAB5B* gene rs1045435, rs11550558, rs705700 and rs11171718 SNPs were associated with PCOS risk, rs1045435 SNP was associated with plasma miR-24, and rs11550558, rs705700, rs11171718 SNPs were correlated to plasma level of miR-320. It is speculated that rs1045435 may be a binding site for miR-24, while rs11550558, rs705700 and rs11171718 may be binding sites for miR-320, which needs further studies to support.

## Abbreviations

BMI: body mass index
CI: 95% confidence interval
FSH: follicle stimulating hormone
GWAS: genome-wide association research
HWE: Hardy–Weinberg equilibrium
LH: luteinizing hormone
miRNA: microRNA
OR: odds ratio
PCOS: polycystic ovary syndrome
PCR: polymerase chain reaction
RAB5B: Ras-related protein Rab-5B
RT-qPCR: reverse transcription fluorescent quantitative PCR
TGF-β: transforming growth factor beta
TSH: thyroid stimulating hormone
